# What the Heart Can(not) Tell: Potential and Pitfalls of Biometric Recognition Methods Based on Photoplethysmography

**DOI:** 10.3390/s25247586

**Published:** 2025-12-14

**Authors:** Lidia Alecci, Matías Laporte, Leonardo Alchieri, Nouran Abdalazim, Silvia Santini

**Affiliations:** Faculty of Informatics, Università della Svizzera Italiana (USI), 6962 Lugano, Switzerland; lidia.alecci@usi.ch (L.A.); matiaslaporte@gmail.com (M.L.); leonardo.alchieri@usi.ch (L.A.); nouran.abdalazim@usi.ch (N.A.)

**Keywords:** photoplethysmography (PPG), biometric recognition, wearable sensors, real-world evaluation, user identification, evaluation guidelines

## Abstract

Human physiological signals collected through wearable devices enable a range of applications, including biometric authentication. Prior studies have demonstrated the potential of using physiological signals to uniquely identify individuals, but their validity in real-world scenarios remains limited. Most existing work relies on controlled experimental settings, small datasets, short-term evaluations, and the absence of unseen-user testing—factors that tend to produce overly optimistic performance estimates. Although recent research highlights the need for broader benchmarking and reproducible protocols, systematic evaluations remain scarce. In this study, we assess the reliability of photoplethysmography (PPG)-based biometric methods. We replicate two published approaches and introduce a feature-based method as a baseline, evaluating all three under multiple conditions. Our results show that while these methods perform well in laboratory datasets, their effectiveness declines substantially in real-world environments, where signal variability, larger user populations, and temporal separation between training and testing challenge current systems. To address these issues, we propose guidelines for the robust evaluation of PPG-based biometrics, emphasizing real-world and longitudinal datasets, temporal splits, unseen-user assessments, and transparent reporting. Although developed for PPG, these recommendations generalize to other physiological biometrics and aim to improve the reliability and reproducibility of future research.

## 1. Introduction

Personal devices—such as smartphones, smartwatches, wearables, or smart rings—have become invaluable tools for collecting physiological data [1], including heart rate [2] and blood pressure [3,4] among others. These devices not only enhance health monitoring but also open new possibilities for authentication systems. Traditional methods, such as passwords and PINs, rely on knowledge-based credentials (“what you know”), making them vulnerable to issues like phishing and theft. In contrast, biometrics—the research field that studies how individuals can be uniquely recognized from their physical, chemical, or behavioral attributes [5]—rely on traits inherently possessed by individuals (“what you are”). Biometrics operates on the foundational assumption that unique patterns or characteristics can reliably distinguish one individual from another. These traits encompass facial features [6], fingerprints [7], and anatomical structures [8,9], as well as eyebrow and the nose-tip features for facial recognition [10], and the structure of the blood vessels underneath the palm skin [9]. Among these, a widely used physiological signal captured by most wearable devices—including the Apple Watch [11], Oura Ring [12], and Samsung Watch [13]—has demonstrated the ability to encode unique characteristics of heart mechanics and pulse [14]. This signal also conveys information about capillary blood vessels [9], skin tone, and body mass index [15]. These features collectively enable the unique characterization of individuals based on data from wrist-worn wearables [16].

While prior research has demonstrated that PPG can be effectively used for biometrics [17,18,19,20], their evaluation methods and the datasets used introduce limitations that can overestimate performance. Specifically, we identify four key factors that impact the validity and generalizability of PPG-based biometric systems: (1) the prevalence of controlled experimental settings, which often fail to capture the variability in physiological data seen in real-world environments; (2) the number of users in the datasets, which may simplify recognition tasks and raise questions about their scalability; (3) the need to address scenarios where new users attempt to authenticate; and (4) the lack of evaluation methods that separate training and test data across distinct time spans, preventing an assessment of model performance on new data collected after an initial training phase. Although these issues are recognized in biometrics research broadly, systematic empirical assessments within the context of PPG-based methods are still lacking. Most studies mention these factors qualitatively [21], but few explicitly measure their individual effect on model reliability.

Three of these four factors stem from dataset limitations, a common issue in the wearable domain due to the challenges of collecting real-world longitudinal data [22,23,24]. To systematically assess these constraints, we leverage the LAUREATE dataset [25], one of the few longitudinal datasets that provide raw PPG data from 44 participants. This dataset enables a more rigorous evaluation of model performance over extended periods, addressing the need for real-world variability and long-term assessment. We replicate two prior studies [26,27] and introduce a feature-based approach that incorporates both time- and frequency-domain characteristics as a baseline. We then assess the impact of dataset size, real-world variability, unseen users, and temporal separation on biometric performance.

Our findings indicate that PPG-based user recognition is unreliable in real-world conditions. Models that achieve a Matthews Correlation Coefficient (MCC) of 0.94 in controlled settings suffer performance drops of up to 0.61 points when tested on real-world data. Specifically, on the LAUREATE dataset, the MCC declines from 0.70 to 0.09 for one method and from 0.94 to 0.44 for another, underscoring the impact of real-world variability on biometric performance. We further show that small dataset sizes artificially inflate results, as models trained on fewer users face a simpler classification task. Moreover, we highlight that the often-overlooked open-set scenario, where new users not present during training attempt authentication, should be considered. Finally, we observe that random train–test splits substantially inflate performance. When the training and test sets are instead separated in time—preventing the model from exploiting short-term temporal similarities—the MCC drops below 0.17. This indicates that the models do not learn stable biometric characteristics but rather rely on transient patterns present within closely sampled data. To support more rigorous evaluation in future work, we also outline a set of guidelines for PPG-based biometrics, emphasizing the importance of adequate dataset size, real-world variability, unseen users, and proper temporal separation.

The main contributions of this paper are as follows:

- A systematic categorization of biometric techniques presented in the literature;
- Replication of prior PPG-based biometric methods and comparative evaluation of their performance under real-world constraints;
- Identification and assessment of factors that may inflate reported biometric performances in the literature;
- Recommendations for an evaluation setup that robustly assesses the performance of biometric models.

The code used to run the analyses presented in this study is available on GitHub: https://github.com/LidiaAlecci/PotentialPitfallsBiometricPPG (accessed on 9 December 2025). The LAUREATE dataset is available upon signing a data-sharing agreement, whereas the BIDMC PPG and Respiratory dataset and the Real-World PPG dataset are publicly accessible.

## 2. Background and Related Work

### 2.1. User Recognition Taxonomy

The reviewed literature uses different terms for user prediction within a dataset: *verification*, *identification*, *authentication*, *re-identification*, and *recognition*. For example, Bianco and Napoletano [28] use *recognition* and *identification* interchangeably; they refer with both terms to a classification task in which a model, given an input, determines which user from the training set it belongs to. However, Biswas et al. [19] implement *identification* using a one-vs.-rest approach, i.e., a separate model is trained for each user to distinguish that specific user from all others. Similarly, Cornelius et al. [29] consider *identification* as the one-vs-rest approach, using *verification* for the classification approach. Lastly, both Almuashi et al. [30] and Peralta et al. [31] in their literature surveys define *identification* as the identification of *n* users, and *verification* as the binary classification task (one-vs-rest).

We categorize biometric research into three tasks: *verification*, *identification*, and *re-identification*, with *recognition* used as the overarching term. This categorization is based on how each system is designed and its intended purpose; a summary of our terminology is provided in Table 1.

In *identification*, the system aims to distinguish an individual from a population, involving a classification task with multiple classes corresponding to *n* possible users, similarly to Piciucco et al. [18]. In *verification*, the system seeks to validate a person’s identity, resulting in a binary classification task where the model discriminates between the user it is trained on and all the others as in Biswas et al. [19]. *re-identification*, first defined in the domain of multi-camera surveillance systems [32], focuses on identifying the same person appearing in non-overlapping or disjoint camera views, accounting for variations in lighting conditions, poses, and camera perspectives. In the field of biometrics, Seok et al. [26] address re-identification using PPG, while Wu et al. [33] address re-identification within video surveillance systems.

The distinction between *re-identification* and *identification* lies in the system’s design. In *re-identification*, all samples are cross-referenced to create pairs from the original pool of samples, and the system determines whether two samples correspond to the same user or not. Since *re-identification* focuses solely on pairwise similarity rather than explicit identity assignment, it can generalize to users never encountered during training. In contrast, *identification* involves a system that, given a sample as input, assigns it to one of the users seen during training. This approach inherently assumes that all possible users are present in the training set. As a result, an *identification* model must be retrained to accommodate new users, whereas *re-identification* remains applicable even when encountering users not seen during training.

### 2.2. Biometrics from PPG

In Table 2, we summarize current state-of-the-art approaches for identification, verification, and re-identification using the PPG signal. While identifying users through PPG is not a new approach, there remains a gap in evaluating solutions within real-world scenarios, where external factors such as noise and emotional variations can affect the signal, and on longitudinal datasets spanning months of recordings.

Piciucco et al. [18] explore biometrics using electrodermal activity (EDA) and PPG signals. The dataset used comprises 17 participants engaged in lectures, with data collected during two separate sessions spaced seven days apart. The study employs convolutional neural networks (CNNs) to extract features from the spectrograms of the EDA and PPG signals. The highest accuracy is achieved using a multimodal approach combining both signals, yielding an average correct identification rate of 98.58%. Similarly, Hinatsu et al. [27] focus on biometric identification using PPG signals but with an emphasis on both heartbeat and respiration-derived features, such as the maximum, minimum, and standard deviation of the pulsatile, non-pulsatile, and respiratory components. Hinatsu et al. [27] extract Mel-Frequency Cepstrum Coefficients from the PPG signals to capture additional physiological characteristics. Their method, tested on a dataset of 46 users, achieves an accuracy of 92.9% using a random forest classifier. Jindal et al. [16] instead opt for a deep learning approach, applying Restricted Boltzmann Machines and Deep Belief Networks for classification. They use the TROIKA dataset containing PPG signals from 12 male users, who were recorded during walking and running activities at various speeds. Preprocessing steps include filtering to remove motion artifacts, segmentation, and feature extraction. The method achieves an accuracy of 96.1% using 10-fold cross-validation. Seok et al. [26] investigate PPG-based biometric authentication using a Siamese 1D convolutional neural network within a re-identification framework, reporting 97.23% accuracy on 35 subjects.

From the results in the mentioned literature, recognizing users across datasets seems feasible. However, we show empirically that prior research tends to overestimate the reliability of their findings for several reasons. For instance, Hinatsu et al. [27] and Jindal et al. [16] evaluate their methods exclusively on datasets collected in controlled laboratory settings, leaving their applicability to real-world environments untested. Although Piciucco et al. [18] utilize a real-world dataset, the study is limited to only 17 users, which restricts the generalizability of the results.

In summary, while the existing literature demonstrates promising results in biometric, several factors suggest that these findings might be overly optimistic. Many studies rely on small sample sizes or controlled environments and lack proper evaluation, raising concerns about their generalizability and scalability in real-world settings. Therefore, in this paper, we critically assess how current PPG-based biometric approaches fail to sustain their effectiveness outside controlled settings. Additionally, we propose guidelines for an evaluation framework that enables future research to design testing setups that more accurately reflect real-world scenarios. While the need to account for environmental variability and unseen users has been discussed in other biometric modalities such as face or fingerprint recognition, their quantitative impact on physiological biometrics remains underexplored.

## 3. Datasets, Algorithms and Evaluation Metrics

Reproducing previous studies is often challenging due to the unavailability of code or datasets. In our case, as shown in Table 2, no implementation code was provided for the majority of relevant prior works, and only a few datasets were publicly available. To determine which studies to reproduce, we established the following criteria: the study must use PPG data and rely on a publicly available dataset. The latter criterion is essential, as access to the dataset allows us to verify the implementation of the methods described in their papers and compare our results with their reported performance.

Therefore, we selected the following two studies for comparison: Hinatsu et al. [27] and Seok et al. [26].

### 3.1. Datasets

#### 3.1.1. BIDMC PPG and Respiration Dataset

The dataset presented by Pimentel et al. [40], hereinafter referred to as the BIDMCPPG dataset, contains PPG signals from 53 adults (median age: 64.81, age range: 19–90+, 32 females), sampled at a frequency of 125 Hz, with each session lasting 480 s. This dataset is used by Hinatsu et al. [27], although their implementation relies on a subset of 46 subjects rather than the full cohort.

#### 3.1.2. Real-World PPG Dataset

The dataset presented by Siam [41], hereinafter referred to as the RWPPG dataset, contains PPG signals from 35 users. Most recordings are collected during a single, long session on the same day for each participant and then divided into 6 s segments [42] sampled at 50 Hz. The dataset is already partitioned into training and test sets: the training set contains approximately 40 samples per participant (1374 samples in total), while the test set includes roughly 20 samples per participant (700 samples in total). Train and test splits, obtained by random selection, are provided by the authors [43]. This dataset is used by Seok et al. [26].

#### 3.1.3. LAUREATE

The LAUREATE dataset [25] was collected during two university courses and includes data from 44 participants: 42 students and two professors.

Each course consisted of biweekly lectures over a 13-week period, totaling 52 recorded sessions. A typical lecture lasted 90 min and followed a frontal teaching format, though some sessions included laboratory activities, quizzes, or exams. In total, the dataset comprises over 1400 h of physiological recordings. For our study, we focus on the 42 students, excluding the professors to prevent artificially inflated results. Professors generally exhibit greater movement during lectures, introducing motion artifacts in the PPG data. This increased variability makes them easier to distinguish from students, thereby facilitating their recognition. To mitigate *device bias*—a key challenge in *user recognition* [44,45]—participants were assigned different devices for each lecture.

We restrict our analysis to data collected during frontal lectures, excluding quizzes, exams, and laboratory sessions. Including these additional sessions substantially increases vigorous movement and device manipulation, introducing severe motion artifacts. Although such artifacts naturally occur in real-world settings, their magnitude strongly depends on device placement—here, the wrist—where even moderate movement can markedly degrade the PPG signal. Because our goal is to evaluate PPG-based authentication under realistic but not extreme conditions, we retain only frontal lecture sessions, where movement is limited yet still naturalistic.

As a result, the final dataset includes 42 participants and comprises around 700 h of recorded data. We selected the LAUREATE dataset for our analysis because it is a longitudinal dataset spanning several months of recordings. By capturing physiological changes influenced by real-world conditions, it provides a unique opportunity to assess how user recognition models handle variability over time.

### 3.2. Algorithms

Hinatsu et al. [27] employ a shallow machine learning technique (i.e., Random Forest), while Seok et al. [26] utilize a deep learning model (i.e., Siamese Neural Network). This selection allows our findings to be generalizable across both paradigms—i.e., both classical machine learning and deep learning approaches. As the original code for both studies is unavailable, we made specific methodological choices in cases of ambiguity or insufficient procedural details. The Supplementary Materials provides further details to support the reproducibility of our analysis in future research. Our goal is not to outperform these models but to use them as representative anchors for evaluating robustness under different data regimes. In addition to replicating their methods, we developed a baseline approach, hereinafter referred to as feature-based method.

#### 3.2.1. Hinatsu et al. [27]’s Method

They propose a PPG-based subject identification method, leveraging features from heartbeat and respiration for continuous authentication. The signal is segmented into 40 s windows and decomposed into three components: AC (high-frequency), RS (mid-frequency for respiration), and DC (low-frequency for blood volume). Feature extraction includes 20 spectral and statistical features from AC and RS, 5 additional features from DC, and MFCC coefficients. A Random Forest classifier is trained using a leave-one-out evaluation, i.e., one data window is held out at a time while the remaining samples are used for training. More implementation details are provided in the Supplementary Materials and in the authors’ original paper paper [27].

#### 3.2.2. Seok et al. [26]’s Method

The authors developed a PPG-based re-identification system using a one-dimensional Siamese neural network. The signal is first segmented into 6 s windows, followed by linear detrending to enhance peak detection. Feature extraction involves peak detection, quadratic spline interpolation for cycle standardization, and multicycle averaging to improve signal consistency. Evaluation is conducted using the predefined train–test split from the RWPPG dataset. More implementation details are provided in the Supplementary Materials and in their paper [26].

#### 3.2.3. Our Feature-Based Method

We extract multiple signal components from the raw PPG signal: Fast Fourier Transform, Welch’s method, Wavelet Daubechies 1, Wavelet Daubechies 4, and Wavelet Symlets 4. A total of 160 features are extracted, comprising 144 time-domain and 16 frequency-domain features, computed across all components. The model we employ is XGBoost. Full implementation details are available in the Supplementary Materials. The evaluation setup and window size are tailored to each task and adjusted based on whether the experiment includes a comparison between feature-based method and the other two algorithms. In comparative cases, the evaluation approach and window size are aligned with those of the other algorithms to ensure a fair and consistent comparison. The specific evaluation procedure for each experiment is detailed before presenting the results. In a re-identification scenario, our feature-based method concatenates the two inputs, which are then fed into the XGBoost model.

### 3.3. Metrics

We report results using both accuracy and balanced accuracy as commonly recommended in the literature [46]. Since accuracy alone is not suitable for unbalanced datasets [47]—as is the case for most of our of our experiments—we also include the Matthews Correlation Coefficient (MCC) [48], a metric that is robust to class imbalance. Although the Matthews Correlation Coefficient (MCC) was originally introduced for binary classification problems [48], it admits a straightforward extension to the multi-class case based on the full confusion matrix, where it still measures the correlation between true and predicted labels. In line with recommendations for imbalanced classification tasks [47], we therefore report the multi-class MCC in our identification experiments, computing it over all users (42 or 53 classes depending on the dataset). This yields a robust performance metric that remains informative even when class distributions are skewed and accuracy alone may be misleading.

Across all experiments, each model is initialized using ten different random seeds. In the tables, we report the standard error as a subscript for each metric, while the plots display the standard error as error bars. When statistical tests are performed, we apply a one-sided Wilcoxon signed-rank test [49], with Bonferroni correction for multiple comparisons [50].

## 4. Results

In this section, we present the results of our study. First, we benchmark the different methods using the datasets used in the literature. We then describe different experiments assessing the factors that, to our understanding, contribute to the overestimation of biometric results in the literature. Rather than pursuing state-of-the-art accuracy, this analysis emphasizes how methodological design choices influence the reported performance of biometric systems. By systematically quantifying the extent of overestimation arising from common evaluation practices, our work complements algorithmic research with an evidence-based perspective on experimental rigor.

### 4.1. Benchmarking

As the first step of our analysis, we try to reproduce two studies [26,27] from the literature whose datasets are publicly available, to create a benchmark of the different methods and datasets. We follow the same evaluation procedure and use the same window size as the original studies. For the BIDMCPPG dataset, we apply a leave-one-out approach—i.e., one test sample is held out at each iteration, and the remaining samples are used for training—and use a 40 s window [27], while for the RWPPG dataset we use the train–test split provided by the dataset authors with a 6 s window [26]. We report the results of the benchmark in Table 3.

Notably, our implementation of Hinatsu et al. [27] achieves higher accuracy compared to the results published in their paper, with an improvement of approximately 0.02 percentage points. Likewise, our re-implementation of Seok et al. [26] attains an accuracy that is 0.01 percentage points higher. These differences may stem from random variation in the results or from minor discrepancies in our re-implementation (see the Supplementary Materials for more information).

Nevertheless, we consider this difference negligible and unlikely to significantly impact our overall conclusions. Our implementation of the method described by Seok et al. [26] achieves the same accuracy reported in their paper. For this method, we also rely on some assumptions to re-implement the original method, described in detail in the Supplementary Materials.

Our feature-based method and our implementations of previous works achieve over 0.89 accuracy, underscoring the relative ease of these tasks under controlled conditions. However, we believe these results may be overestimated for several reasons. First, both datasets are collected in laboratory settings where physiological data, though still variable, may not reflect the natural fluctuations encountered in real-world environments [51]. Second, these works do not account for the challenge of unseen users attempting to authenticate into the system, which is crucial for real-world applications. Finally, these methods have not been evaluated on temporally disjoint data, leaving their long-term effectiveness uncertain.

To address these limitations, in Section 4.2, we further test both our feature-based method and our implementation of Hinatsu et al. [27] and Seok et al. [26] methods on a real-world, longitudinal dataset (LAUREATE) to better evaluate their applicability in practical, everyday scenarios.

### 4.2. Laboratory vs. Real-World Scenarios

A significant number of experiments are conducted in controlled laboratory environments [16,27,38], where researchers attempt to isolate specific variables. However, these controlled settings do not necessarily translate to real-world conditions, and often fail to capture the inherent variability in human physiology [52]. Physiological responses are not only highly variable between individuals but can also fluctuate within the same individual depending on the context and even time of day [53,54]. This situation not only complicates the consistency and reproducibility of lab experiments but also challenges the assumption that the methods developed and tested in controlled environments are applicable to real-world settings.

To evaluate how these methods perform in a real-world scenario, where users are exposed to external stimuli such as emotions and movement, we test our feature-based method and our implementation of the two previously used methods on the LAUREATE dataset [25]. To isolate and assess only the impact of the real-world environment, we keep all other relevant factors as close as possible to the previous setting.

First, for each user in the LAUREATE dataset, we only use data from one day—namely, the user’s first day in the dataset. Second, we also segment the data as in the original studies, i.e., 40 s windows for identification (our implementation of Hinatsu et al. [27]) and 6 s windows for re-identification (i.e., Seok et al. [26]). Third, we also match the number of samples, using 12 samples per user for identification and 60 for re-identification. Furthermore, to ensure good quality readings (i.e., comparable to laboratory studies) we also discard the first and last 5 min of each session, ensuring samples are taken while students are engaged in class, avoiding noise from putting on or removing the device. We refer to this dataset as “LAUREATE one-day”. Finally, we use the same evaluation procedure as in the previous experiment: leave-one-out approach for identification, and a random 66%-34% train–test split for re-identification.

As shown in Table 4, the accuracy obtained on the LAUREATE dataset—collected in the real-world—is statistically lower than the results achieved in laboratory settings (reported in Table 3). For the identification task, the MCC comparison between the laboratory dataset and the LAUREATE dataset reveals a disparity of up to 0.50 MCC points between the two settings— Hinatsu et al. [27] on their laboratory dataset (0.94) versus the same method on the real-world LAUREATE dataset (0.44). This decline is even more pronounced in the re-identification task, where the MCC difference between the laboratory setting and the LAUREATE dataset reaches up to 0.61 MCC points—0.70 using the Seok et al. [26] method on the laboratory dataset versus 0.09 on the real-world LAUREATE dataset. This underscores how real-world conditions, where various factors can degrade physiological data quality, add a level of complexity that methods tested only on laboratory data may struggle to manage effectively.

We emphasize that relying solely on accuracy for the re-identification task is misleading, as the task is inherently imbalanced, with the positive class significantly underrepresented in the test set. As Chicco and Jurman [47] highlight, traditional metrics often fail to provide a meaningful assessment in such cases. Specifically, although our feature-based method for the re-identification task on the LAUREATE one-day dataset achieves an accuracy of 0.82 and a balanced accuracy of 0.76, these values are misleadingly high, as the MCC is only 0.20, indicating poor overall performance.

In the next two experiments, we focus on the best-performing model and the task that yields the highest accuracy. Therefore, we select the identification task and use only our feature-based method.

### 4.3. Impact of the Number of Users

The datasets used by Hinatsu et al. [27] and Seok et al. [26] include 53 and 35 participants, respectively. In contrast, most studies summarized in Table 2 involve approximately 20 users. While training and testing methods on a limited number of participants already poses generalization issues in domains like human activity recognition [55], we argue that this challenge is even more critical in the biometric domain. In this context, the number of users determines the number of outputs of the model. Consequently, having fewer users increases the probability of achieving a correct prediction by chance.

To evaluate the impact of the number of participants on prediction performance, we conduct the following experiment. Using only the first day of data for each participant from the LAUREATE dataset, we select a 30 min segment from the middle of the lecture to ensure data quality and maintain balance among all users. This approach aligns with the methodology used by Piciucco et al. [18] in a similar scenario. Then, we segment the data into 40 s windows, and randomly select 30% as the test set. For this experiment, we use our feature-based method, which demonstrates the highest performance in the closely related previous task (reported in Table 4).

**Figure 1:**
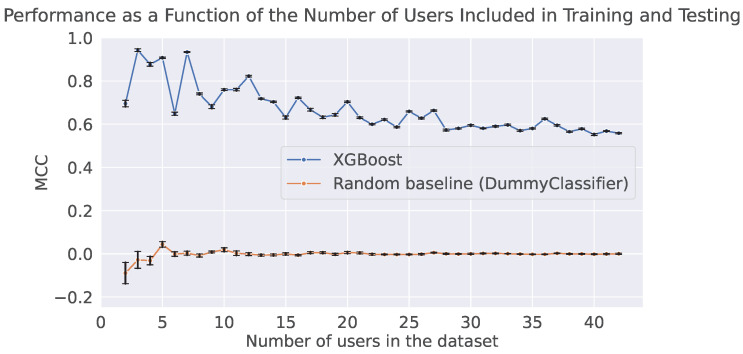
MCC of the user identification model as a function of the number of users in the dataset. The MCC of our feature-based method is shown in blue, while the random baseline, shown in orange, makes predictions uniformly at random from the list of unique classes observed in the training set. The error bars represent the standard error across the ten runs.

As shown in Figure 1, the performance of the model is inversely proportional to the number of users in the dataset. This finding mirrors the results observed by Sugrim et al. [56] in different domains, such as localization predictions and sports performance. Specifically, in our case, MCC is nearly perfect with only two users but declines rapidly until reaching eighteen users. By plotting these results, we can also assess whether the model remains stable as the number of users increases. Notably, with our model applied to the LAUREATE one-day dataset, performance tends to stabilize around 35 users. For comparison, we also report a random baseline alongside our feature-based method.

### 4.4. Unseen Users at Testing Time

A key limitation in many existing studies ([18,19,34]) is their focus on verifying users who have already been registered and included in the system’s training data (here referred to as *known* or *previously seen* users). However, these studies often overlook the system’s vulnerability when faced with users who attempt authentication but were not part of the initial training data. In some cases, training and testing may indeed involve the same set of users, for example, in scenarios where users undergo an initial authentication step and the system’s only role is to assign permissions or manage access within this known group. However, systems designed for open environments must account for the possibility of encountering unknown users. Since it is infeasible to train an authentication system on every potential user, these systems must develop robust strategies to effectively reject unseen users, risking otherwise to incorrectly authenticate unauthorized users. This issue is particularly problematic in machine learning-based identification methods, where models can only predict from a fixed set of classes corresponding to the users seen during training. When an unseen user attempts to authenticate, the model is forced to assign them to one of the known classes, potentially granting access to unauthorized individuals.

To illustrate how the number of unseen users affects a system’s performance, we use the same setup as described in Section 4.3. We select data from the first day for each participant in the LAUREATE dataset, extracting a 30 min segment from the middle of the lecture on that day and we segment the data into 40 s windows.

**Figure 2:**
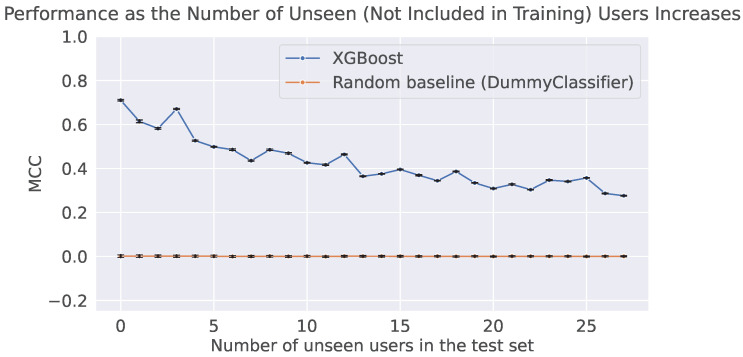
MCC of the user identification model as the number of unseen users in the test set varies. The MCC of our feature-based method is shown in blue, while the random baseline, shown in orange, makes predictions uniformly at random from the list of unique classes observed in the training set. The error bars represent the standard error across the ten runs.

To analyze how performance changes as the number of unseen users increases, we start with 15 users in the training set, leaving up to 27 users as unseen cases for evaluation. As shown in Figure 2, the performance of the model decreases as new unseen users are added to the test set. This decline highlights the inability of the model to effectively handle unseen users, causing it to incorrectly assign them to one of the existing classes. Consequently, the number of false positives increases in direct proportion to the number of unseen user samples and, therefore, the number of unseen users. For comparison, we also report a random baseline alongside our feature-based method.

Notably, there are several approaches for developing a model that can handle unseen users. Techniques like open-set recognition [57]—where the model is designed to determine whether a given input belongs to a known class or an unknown one—and anomaly detection algorithms can be used to identify and reject unfamiliar users. Additionally, instead of focusing on identification, alternative methods such as verification, where a unique model is created for each user to distinguish that user from others, or re-identification, where the input is compared with all samples encountered during training and either matched with the correct identity or rejected, can also be considered.

### 4.5. Temporal Separation of Training and Test Data

A common limitation in many machine learning studies involving time-series data, such as in [26,58], is the lack of evaluation methods that separate training and test data across distinct time spans. Without this separation, performance metrics are usually inflated [59], as models tend to leverage short-term sequential patterns rather than genuinely learn relevant predictive features. In contrast, evaluating the model on data from a later, separate period provides a measure of its ability to generalize and predict beyond immediate, localized patterns.

To assess how performance is affected when modifying the evaluation procedure (i.e., from a random split of training and test sets to a more robust scenario), we split the LAUREATE dataset along the temporal dimension. We use the first half of the dataset for training, while we reserve the latter half for testing. We then apply the same models used in Section 4.2, including our method and our implementation of those from Hinatsu et al. [27] and Seok et al. [26]. To compare the results of this new experiment with those from Section 4.2, which uses only one day of data from the LAUREATE dataset, we select a subset that allows for a similar training set size. Specifically, for the identification task, we extract 24 samples per user. This allows for an even split of 12 samples for training and 12 for testing, resulting in a training set size that matches that of the previous experiment. Similarly, for the re-identification task, we select 120 samples per user. To maintain balance among users, we evenly distribute the samples across the days when each participant is present. For example, if a participant attended 12 days and we need to extract 24 samples per user, we select 2 samples from each day. The sampled windows are randomly chosen from all 30 min lecture segments, centered within the lecture as described in Section 4.3.

As shown in Table 5, performance drops significantly when training and test sets are separated along the temporal axis. In this scenario, the MCC is 0.07 and 0.08 for the identification task and 0.02 and 0.06 for the re-identification task, indicating that the model fails to reliably distinguish users. This performance decline can be attributed to two main factors. First, the model struggles to identify users when their individual patterns differ due to varying daily conditions, such as emotions or stress on a given day [60]. Second, while we reduce the number of samples to ensure a fair comparison between results, this reduction may impair the learning capabilities of the model. To isolate the effect of the first factor, we rerun the experiment without restricting the dataset size. The results are reported in Table 6.

Using the entire LAUREATE dataset does not present scalability issues for identification tasks; however, this is not the case for re-identification scenarios as detailed in the Supplementary Materials. Specifically, in the setup used by Seok et al. [26], where all possible combinations of samples are generated as input for the Siamese Neural Network, increasing the number of samples in the dataset leads to an exponential increase in the number of pairs. While it might be feasible to manage this growth by exploring new solutions, such as subsampling pairs to reduce redundancy (e.g., by excluding positive pairs from the same user collected on the same day), doing so would significantly alter the model initially presented by Seok et al. [26]. Therefore, we opt not to implement such changes and instead present only the results of the identification task in Table 6. We believe this decision does not compromise the overall findings, as the re-identification task demonstrates limited effectiveness in handling a real-world dataset like LAUREATE as shown in Table 4 and Table 5.

Comparing Table 5 and Table 6 reveals a performance improvement in the models, highlighting the importance of having more samples, especially when users must be recognized under varying conditions. While the results improve slightly, with our feature-based method achieving an MCC of 0.16, the models still struggle with user recognition. We show empirically that a longitudinal approach is essential for testing biometric models. In real-world scenarios, training typically occurs during an initial time period, followed by testing on future data with only occasional updates. Therefore, randomly separating training and test sets does not accurately reflect real-world conditions. Given that many current methods struggle to identify users effectively in these situations, we advocate for the development of new strategies and models that can better address these challenges.

## 5. Guidelines for Evaluating Methods for Biometric Systems

To ensure that biometric systems are effective, scalable, and capable of generalizing to real-world conditions, it is essential to establish rigorous evaluation setups. In this section, we outline key recommendations for evaluating biometric methods. Although our findings focus specifically on methods using PPG data, we believe they generalize to other signals that exhibit similar characteristics, e.g., high variability due to factors such as emotional state or physical activity. Some of these guidelines are derived from the findings of this study, while others represent general best practices [61].

The guidelines below focus on dataset diversity, robust evaluation setups, appropriate metrics, and reproducibility. Implementing these recommendations enables researchers and practitioners to produce more reliable and replicable results that generalize to real-world conditions.

- **Dataset**
  – **Real-World Data**: Use datasets capturing real-world conditions, to avoid the overestimation of model performance typically found in controlled, laboratory environments.
  – **Longitudinal Data**: Select datasets comprising recordings from multiple sessions over extended time spans. This approach captures real-world variability and enables the evaluation of model robustness over time. For instance, tracking performance across sessions helps assess model degradation and stability under real-world conditions.
  – **Demographic diversity**: In addition to real-world and longitudinal data, biometric evaluations should account for the demographic composition of the population being modeled. The datasets used in our study—and much of the PPG literature—primarily involve young adult participants. This limited representation overlooks the fact that patterns identifiable to a young population may not be the same for an older population.
  – **Health condition diversity**: Biometric systems should also consider variability arising from differences in health status. Studies relying on small, homogeneous cohorts may inadvertently inflate performance. For example, Gill et al. [62] show that older patients with permanent atrial fibrillation and heart failure monitored with consumer wearables exhibit marked intra- and inter-individual variability in heart-rate dynamics.
  – **Number of Users**: A large portion of available datasets includes fewer than 35 users (e.g., [17,18,34]). However, it is essential to demonstrate that this number is sufficient for reliable evaluation. Future studies should report model performance as a function of the number of users to assess how metrics vary with sample size, similarly to what is shown in Figure 1. If performance stabilizes as the user count increases, it may indicate that the model is likely to generalize well, even with a larger user base.
- **Evaluation Setup**
  – **Temporal Split**: Separate training and test sets from distinct time spans rather than using random splits. This approach prevents the model from leveraging short-term sequential patterns and simulates real-world usage more accurately.
  – **Testing with Unseen Users**: Evaluate how models respond to new, unseen users in the test set. Include an “unseen user” evaluation to ensure that the model does not mistakenly classify unauthorized users.
  – **Balanced Evaluation**: For unbalanced datasets, choose metrics like Matthews Correlation Coefficient (MCC) to ensure evaluation results remain reliable even with label imbalance.
- **Transparency and Reproducibility**
  – **Detailed Documentation**: Provide detailed descriptions of preprocessing steps, hyperparameters, and model architecture to facilitate reproducibility.
  – **Open-Source Code**: Make the code used for evaluation and model implementation publicly available. Open-source code promotes transparency, enables reproducibility, and allows other researchers to verify findings and build upon the work, fostering progress in the field.
  – **Open-Source Dataset**: Use or release open-source datasets to promote replicability and broader model testing. If sharing the entire dataset is not feasible due to ethical considerations, consider providing controlled access through a data-sharing agreement to ensure legal and ethical compliance.

By following these recommendations, future studies can establish more robust, realistic, and generalizable evaluation setups for biometric methods. To demonstrate the current relevance of these guidelines, Table 7 presents whether each paper mentioned in the related work section adheres to the proposed guidelines. For the assessment of qualitative guidelines, we define adherence as follows: for the guideline “number of users”, adherence is met if the study includes more than 35 participants; for “demographic diversity”, adherence is met if the study includes participants from at least two distinct demographic groups (in this case, we assessed age and sex, defining age diversity as the presence of participants whose ages differ by at least 50 years; for “health condition diversity”, adherence is met if the study includes both healthy individuals and those with a diagnosed clinical condition; and for “longitudinal data”, adherence is met if data are collected on multiple days for all participants. Although “detailed documentation” is difficult to fully assess, we consider a paper compliant if its code is available and well-commented. If the code is unavailable, the paper should instead provide sufficient detail to enable the reconstruction of preprocessing steps and the implementation of the model. Finally, adherence to “balanced evaluation” is achieved if the test set is balanced, or if, in cases of imbalanced data, the MCC is reported. If no information is provided, adherence for that parameter is not met.

## 6. Limitations and Future Works

Our study aims to highlight how some of the previous works tend to report results that are not sustained when applied to more real scenarios. This inadvertent overestimation is caused by limitations in the used datasets and chosen evaluation procedures. In particular, we identify four main factors contributing to these overestimated results, and study their impact using both a feature-based method developed by us and two other methods from the literature. While our study does not center on methodological advancement, it nonetheless contributes empirically to ongoing discussions about evaluation standards in physiological biometrics. The insights provided here can guide the design of future, more advanced methods. Nevertheless, our work has several limitations, which are discussed here.

Firstly, the longitudinal dataset we use was collected using the Empatica E4, a medical-grade device that is CE-certified in Europe and has been successfully used in a wide range of studies and tasks [63,64]. However, most previous works, including the studies selected for our analysis, employed different sensors, making it unclear whether the observed performance degradation is solely due to the transition from controlled laboratory conditions to more variable real-world settings, or whether differences in the sensing devices also play a role. Future work could focus specifically on assessing the differences in signal quality and model performance when collecting data from the same users in both laboratory and real-world conditions.

Secondly, although our dataset includes multiple users with recordings over several days, all users come from the same university and belong to a similar age range. Future research could explore how increasing diversity in the dataset—across demographics and environments—might further impact biometric performances.

Additionally, among the four identified limitations, only one stems from algorithm design, while the remaining three arise from dataset constraints. Thus, although we evaluate these limitations in the context of biometrics, they may also affect other systems, such as human activity recognition.

## 7. Conclusions

In this study, we critically evaluated the reliability of biometric methods based on photoplethysmography (PPG) and proposed guidelines to enhance the robustness and generalizability of future research in this domain. By replicating prior work and developing a feature-based method as baseline, our findings reveal that, while existing methods show promise in controlled laboratory settings, they often face significant challenges in real-world applications. Different factors such as small sample sizes, the lack of longitudinal data, or testing protocols that do not account for unseen users, lead to an overestimation of model performance which may hinder the applicability of these systems in practical environments.

We propose a set of guidelines to address these challenges, emphasizing the need for larger and more diverse datasets that reflect the variability encountered in real-world use. This includes leveraging longitudinal data collected over multiple days and conditions to assess model stability over time. We also highlight the importance of demonstrating that the approaches remain valid and stable as the number of users increases, ensuring scalability. Additionally, we recommend implementing temporal splits for training and testing to prevent models from relying on short-term patterns that may not generalize well. Furthermore, testing model performance on unseen users is crucial, as it addresses potential security vulnerabilities, enhancing robustness against unauthorized access. We also recommend transparent reporting practices—such as detailed documentation of preprocessing steps, model parameters, and open access to code and datasets—essential for ensuring reproducibility and advancing the field. Finally, in scenarios with class imbalance, we advocate for metrics like the Matthews Correlation Coefficient, which provides a more reliable evaluation in imbalanced conditions compared to traditional metrics.

By demonstrating and quantifying well-known pitfalls within a reproducible framework, our proposed guidelines aim to create more robust evaluation setups, helping future research to develop biometric systems that are not only effective in controlled settings but also resilient and applicable in the variability of daily life. As the field progresses, we hope that these recommendations will contribute to the creation of biometric systems that are both reliable and adaptable, enabling their use across a wide range of conditions.

## Figures and Tables

**Table 1:** Comparison of *identification*, *verification*, and *re-identification* terms.

Task	Objective	Input	Output	Example
identification	Distinguish an individual from a population (i.e., a classification task).	A single input for classification.	*n* possible outputs, each class corresponding to one user.	An authentication system where *n* possible individuals can log in, each potentially having different access rights or permissions.
verification	Validate a person’s identity (i.e., a binary classification task where the model only has to discriminate between the user it was trained on and all others).	A single input for classification.	A binary output, where 1 indicates the target user and 0 denotes a non-target user.	The system verifies whether a person is who they claim to be.
re-identification	Recognize and match individuals across different measurements and/or conditions.	Two inputs for comparison.	Depending on the implementation, either a binary outcome or a similarity score.	Understanding if two users from two different systems are the same user.

**Table 2:** Overview of state-of-the-art approaches for biometric recognition using PPG. ACC represents accelerometer, ECG denotes electrocardiogram, and EDA stands for electrodermal activity. EER denotes Equal Error Rate, AC denotes Accuracy, CIR stands for Correct Identification Rate, and AUC denotes the area under the ROC Curve. The symbols ✓ and ✗ indicate whether the corresponding item is included or not, respectively. Publicly available datasets marked with † require signing a written agreement.

Study	Setting	Signals	Goal	Users	Results	Data Collection	Public	
							Data	Code
Piciucco et al. [18]	Real-world	PPG	Identification	17	CIR = 98.6%	3 weeks	✗	✗
Everson et al. [17]	Laboratory	PPG	Verification	12	AC = 96%	5 min	✗	✗
Hinatsu et al. [27]	Laboratory	PPG	Identification	46	AC = 92%	8 min	✓	✗
Yaacoubi et al. [34]	Laboratory	ECG and PPG	Identification	12	AC = 94%	5 min	✗	✗
Biswas et al. [19]	Laboratory	PPG	Verification	20	AC = 96%	5 min	✗	✗
Liu et al. [35]	Laboratory	PPG and ACC	Identification	20	AC = 98%	5 min	✗	✗
Reşit Kavsaoğlu et al. [36]	Laboratory	PPG	Re-identification	30	AC = 87.2%	Unspecified	✗	✗
Jindal et al. [16]	Laboratory	PPG	Identification	12	AC = 96.1%	5 min	✗	✗
Blasco and Peris-Lopez [37]	Laboratory	ECG, PPG and EDA	Verification	25	EER = 0.019	20 min	✓	✓
Yadav et al. [20]	Laboratory	PPG	Identification	42	EER = 0.46%	8 min	✓ †	✗
				32	EER = 2.11%	40 min	✓ †	✗
				34	EER = 5.88%	13 min	✓ †	✗
Seok et al. [26]	Real-world (limited mobility)	PPG	Re-identification	35	AC = 97.2%	6 s	✓	✗
Luque et al. [38]	Laboratory	PPG	Verification	43	AUC = 86%	1 min	✓ †	✗
				20	AUC = 83%	5 min	✗	✗
Alam [39]	Real-world	PPG, EDA and ACC	Re-identification	5	AC = 69.6%	7 days	✗	✗
				8	AC = 67.1%	20 min	✗	✗
				22	AC = 66.4%	3 h	✗	✗
				28	AC = 65.6%	7 months	✓	✗

**Table 3:** Results of our feature-based method and the two prior works, along with their original results and the outcomes of our re-implementation of their models. MCC denotes the Matthews’s correlation coefficient, and BA stands for balanced accuracy. The random baseline predicts uniformly from the unique classes in the training set. For BIDMCPPG, there are 53 unique classes, whereas RWPPG uses a Siamese Neural Network with two unique classes, where a pair of inputs can either be the same (1) or not (0). “Their method” in the table refers to the approaches from Hinatsu et al. [27] for BIDMCPPG and Seok et al. [26] for RWPPG. “Paper” refers to the results reported in the respective papers, which do not include standard deviations or error estimates. The standard error across ten runs is reported as a subscript.

Dataset	Our Feature-Based Method			Their Method				Random Baseline		
				Paper	Our Implementation					
	Accuracy	BA	MCC	Accuracy	Accuracy	BA	MCC	Accuracy	BA	MCC
BIDMCPPG [40]	$0.89_{0.01}$	$0.89_{0.01}$	$0.89_{0.01}$	0.92	$0.94_{0.01}$	$0.94_{0.01}$	$0.94_{0.01}$	$0.02_{0.00}$	$0.02_{0.00}$	$0.00_{0.00}$
RWPPG [41]	$0.98_{0.01}$	$0.97_{0.01}$	$0.76_{0.01}$	0.97	$0.98_{0.01}$	$0.96_{0.01}$	$0.70_{0.01}$	$0.50_{0.01}$	$0.50_{0.01}$	$0.00_{0.01}$

**Table 4:** Comparison of results on the LAUREATE real-world dataset (one-day subset) and on laboratory datasets. LAUREATE is randomly subsampled to match the amount of data available in BIDMCPPG (identification) and RWPPG (re-identification). For re-identification, we follow the train–test split of the original study and repeat each experiment ten times; results are reported as means with standard errors as subscripts. Laboratory–dataset results (from Table 3) are shown for direct comparison. MCC = Matthews correlation coefficient; BA = balanced accuracy. The random baseline predicts uniformly over the classes (42 for identification; two for re-identification). To assess whether performance on LAUREATE is statistically lower than on laboratory datasets, we report Bonferroni-corrected *p*-values [50] (Wilcoxon one-sided signed-rank test [49]), excluding the random baseline.

Task	Method	LAUREATE One-Day			Laboratory Dataset			MCC
		Accuracy	BA	MCC	Accuracy	BA	MCC	*p*-Value
**identification**	Hinatsu et al. [27]	$0.46_{0.01}$	$0.46_{0.01}$	$0.44_{0.01}$	$0.94_{0.01}$	$0.94_{0.01}$	$0.94_{0.01}$	<0.05
	Our feature-based method	$0.59_{0.01}$	$0.59_{0.01}$	$0.58_{0.01}$	$0.89_{0.01}$	$0.89_{0.01}$	$0.89_{0.01}$	<0.05
	Random baseline	$0.02_{0.00}$	$0.02_{0.00}$	$0.00_{0.00}$	$0.02_{0.00}$	$0.02_{0.00}$	$0.00_{0.00}$	N/A
**re-identification**	Seok et al. [26]	$0.70_{0.02}$	$0.63_{0.01}$	$0.09_{0.01}$	$0.98_{0.01}$	$0.96_{0.01}$	$0.70_{0.01}$	<0.05
	Our feature-based method	$0.82_{0.01}$	$0.76_{0.01}$	$0.20_{0.01}$	$0.98_{0.01}$	$0.97_{0.01}$	$0.76_{0.01}$	<0.05
	Random baseline	$0.50_{0.01}$	$0.50_{0.01}$	$0.00_{0.01}$	$0.50_{0.01}$	$0.50_{0.01}$	$0.00_{0.01}$	N/A

**Table 5:** Comparison of results on the LAUREATE dataset using a longitudinal split (train on the first half of the semester, test on the second) versus a one-day data with a random split. To match training size, the longitudinal data are randomly subsampled to the same amount used in the one-day setup. One-day results (from Table 4, under “LAUREATE one-day”) are repeated here for convenience. MCC = Matthews correlation coefficient; BA = balanced accuracy. The random baseline predicts uniformly over the classes (42 for identification; 2 for re-identification). Results are averaged over ten runs, with standard errors as subscripts. To assess whether longitudinal performance is statistically lower than one-day performance, we report Bonferroni-corrected *p*-values [50] (Wilcoxon one-sided signed-rank test [49]), excluding the random baseline.

Task	Method	1st Half Training 2nd Half Test (Subsample Data)			One-Day			MCC *p*-Value
		Accuracy	BA	MCC	Accuracy	BA	MCC	
**identification**	Hinatsu et al. [27]	$0.10_{0.01}$	$0.10_{0.01}$	$0.07_{0.01}$	$0.46_{0.01}$	$0.46_{0.01}$	$0.44_{0.01}$	<0.05
	Our feature-based method	$0.10_{0.01}$	$0.10_{0.01}$	$0.08_{0.01}$	$0.59_{0.01}$	$0.59_{0.01}$	$0.58_{0.01}$	<0.05
	Random baseline	$0.02_{0.01}$	$0.02_{0.01}$	$0.00_{0.01}$	$0.02_{0.00}$	$0.02_{0.00}$	$0.00_{0.00}$	N/A
**re-identification**	Seok et al. [26]	$0.56_{0.01}$	$0.53_{0.01}$	$0.02_{0.01}$	$0.70_{0.02}$	$0.63_{0.01}$	$0.09_{0.01}$	<0.05
	Our feature-based method	$0.08_{0.01}$	$0.09_{0.01}$	$0.06_{0.01}$	$0.82_{0.01}$	$0.76_{0.01}$	$0.20_{0.01}$	<0.05
	Random baseline	$0.03_{0.01}$	$0.03_{0.01}$	$0.00_{0.01}$	$0.50_{0.01}$	$0.50_{0.01}$	$0.00_{0.01}$	N/A

**Table 6:** Results on the full LAUREATE dataset for the identification task, using a temporal split where the model is trained on the first half and evaluated on the second half. MCC denotes the Matthews’s correlation coefficient, and BA stands for balanced accuracy. The random baseline is constructed to make predictions uniformly at random from the list of unique classes observed in the training set, which consists of 42 classes in this case. The standard error across ten runs is reported as a subscript.

Method	1st Half Training 2nd Half Test		
	Accuracy	BA	MCC
Hinatsu et al. [27]	$0.16_{0.01}$	$0.14_{0.01}$	$0.13_{0.01}$
Our feature-based method	$0.19_{0.01}$	$0.17_{0.01}$	$0.16_{0.01}$
Random baseline	$0.02_{0.01}$	$0.02_{0.01}$	$0.00_{0.01}$

**Table 7:** Evaluation of adherence to the proposed guidelines across reviewed studies. The symbols ✓ and ✗ indicate whether a study complies with or does not comply with a given guideline, respectively.

Guidelines Adherence														
Dataset	Real-world data	✓	✗	✗	✗	✗	✗	✗	✗	✗	✗	✗	✗	✓
	Longitudinal data	✓	✗	✗	✗	✗	✗	✗	✗	✗	✗	✗	✓	✓
	Demographic diversity	✗	✗	✗	✗	✗	✗	✗	✗	✗	✗	✗	✗	✗
	Health condition diversity	✗	✗	✗	✗	✗	✗	✗	✗	✗	✗	✗	✗	✗
	Number of users >35	✗	✗	✓	✗	✗	✗	✗	✗	✗	✓	✓	✗	✗
Evaluation setup	Temporal split	✓	✗	✗	✗	✗	✗	✗	✗	✓	✗	✗	✗	✗
	Testing with unseen users	✗	✗	✗	✗	✗	✗	✗	✗	✗	✗	✗	✓	✗
	Balanced evaluation	✓	✗	✓	✗	✗	✗	✗	✗	✓	✗	✗	✗	✗
Transparency and reproducibility	Detailed documentation	✗	✓	✗	✗	✓	✓	✗	✓	✓	✗	✗	✗	✗
	Open-source code	✗	✗	✗	✗	✗	✗	✗	✗	✓	✗	✗	✗	✗
	Open-source dataset	✗	✗	✓	✗	✗	✗	✗	✗	✓	✓	✓	✓	✗
	Studies	[18]	[17]	[27]	[34]	[19]	[35]	[36]	[16]	[37]	[20]	[26]	[38]	[39]

## Data Availability

All three datasets used in this study are publicly accessible to the research community. The BIDMC PPG and Respiration Dataset is available for download from PhysioNet (https://physionet.org/content/bidmc/1.0.0/ (accessed on 9 December 2025)). Real-World PPG Dataset is available for download from Mendeley Data (https://data.mendeley.com/datasets/yynb8t9x3d/1 (accessed on 9 December 2025)). Access to the LAUREATE dataset requires signing a data-sharing agreement. Interested researchers should contact the corresponding author of Laporte et al. [25].

## Supplementary Materials

The following supporting information can be downloaded at: https://www.mdpi.com/article/10.3390/s25247586/s1, The Supplementary Materials provide additional implementation details of the reimplemented methods from the literature and of our feature-based approach. Figures S1 and S2 show the processing pipelines of the methods by Hinatsu et al. [27] and Seok et al. [26], respectively. Figures S3 and S4 compare intermediate processing steps between our reimplementation of Seok et al. [26] and their original study. Figures S5 and S6 illustrate our feature-based identification and re-identification pipelines. References [26,27,41,65,66,67,68,69,70,71,72,73,74,75,76] are cited in Supplementary Materials.
