# Supplementary material for: What the Heart Can(not) Tell: Potential and Pitfalls of Biometric Recognition Methods Based on Photoplethysmography
Source: Sensors (Basel). 2025 Dec 14;25(24):7586. doi: 10.3390/s25247586 (PMC12736706; doi:10.3390/s25247586)
Supplement: Supplementary file 1 [file sensors-25-07586-s001.zip › sensors-4017081-supplementary.pdf]

Supplementary Materials: What the Heart Can(not) Tell: Potential and Pitfalls of Biometric Recognition Methods Based On Photoplethysmography

Lidia Alecci , Matías Laporte , Leonardo Alchieri , Nouran Abdalazim and Silvia Santini *

1. Hinatsu et al. work

Figure S1 illustrates the complete feature extraction and classification workflow proposed by Hinatsu et al. [1].

1.1. Description of the approach

The signal is divided into 40-second segments and decomposed into three components: AC, DC and RS. The AC (pulsatile component) corresponds to the high-frequency component associated with the arterial pulse and is extracted using a bandpass filter with cutoff frequencies of 0.5–5 Hz. The DC (non-pulsatile component) represents the low-frequency component related to overall blood volume and is obtained using a low-pass filter with a cutoff frequency of 0.1 Hz. The RS (respiratory component) corresponds to the mid-frequency component associated with respiratory activity and is isolated using a bandpass filter with cutoff frequencies of 0.1–0.5 Hz.

Features are then computed for each component to characterize physiological patterns. For AC and RS, the extracted features include the maximum, minimum, range, and standard deviation. These four features are also computed from the time derivative of each component. Additionally, the maximum value of the power spectrum and its corresponding frequency are extracted, resulting in a total of 20 features. For DC, the extracted features include the maximum, minimum, range, standard deviation, and mean, leading to a total of five features. Furthermore, Mel-Frequency Cepstral Coefficients (MFCCs) are computed, yielding thirteen-dimensional feature vectors.

The classification model is a Random Forest classifier, evaluated using a leave-one-out cross-validation approach. In each iteration, a single sample is reserved for testing, while the remaining samples are used for training. This procedure ensures that the model is tested on every available sample.

1.2. Issues in the reimplementation

In reimplementing the work of Hinatsu et al. [1], we encountered several ambiguities and made the following choices to address them.

One of the first challenges is the decomposition of the signal into AC, DC, and RS components. The authors did not specify whether they used a particular algorithm or performed the decomposition manually, and no formula was provided. Therefore, we applied a standard approach [2,3], decomposing the signal into AC, DC, and RS components using a 4th-order Butterworth filter as implemented in the SciPy library [4][1].

Another issue arose with the time derivative formula provided, which was written as

$$v[n] = v[n+1]–v[n–1]$$

[1] https://docs.scipy.org/doc/scipy/reference/generated/scipy.signal.butter.html

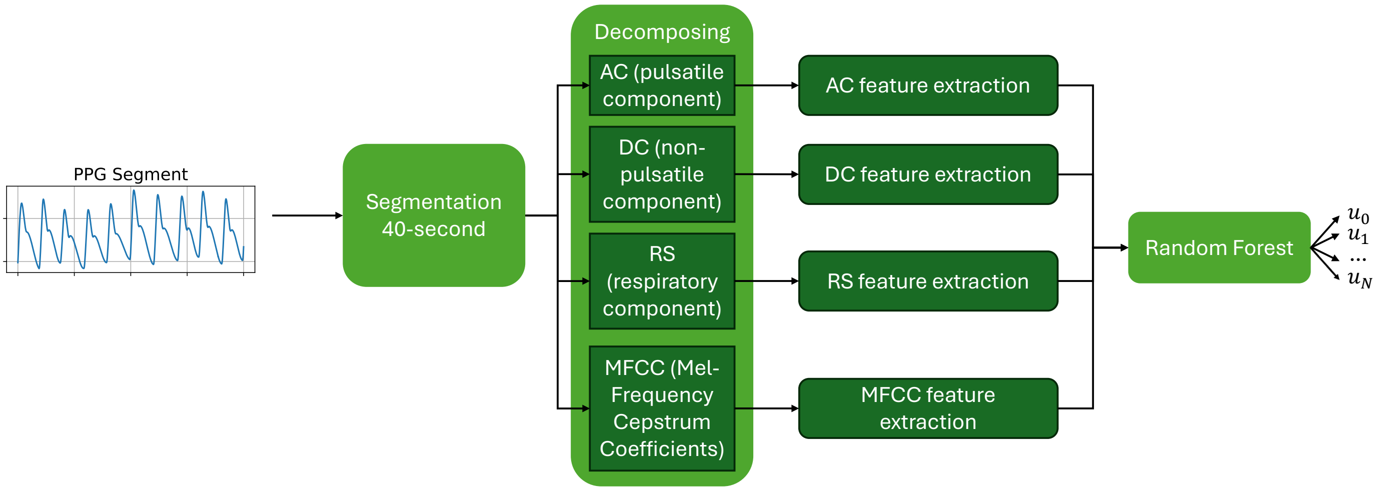

Figure S1. Processing pipeline of the method by Hinatsu et al. [1]. The raw photoplethysmography (PPG) signal is divided into 40-second segments and decomposed into three components: a pulsatile component (AC), a non-pulsatile component (DC), and a respiration-related component (RS). For each component, different features are extracted, together with Mel-Frequency Cepstral Coefficients (MFCCs). All features are then used to train a Random Forest classifier to identify the user.

This formulation seemed unusual, as a more typical approach would be

$$v[n] = v[n] – v[n–1]$$

. Despite this discrepancy, we chose to implement the formula exactly as specified (i.e., the first one) to remain consistent with the original study.

For calculating the power spectrum, the authors described a method involving Hamming points and discrete frequency, but its details were limited. To streamline this process, we applied the Fast Fourier Transform (FFT), as implemented in the `numpy` library[2], to compute the power spectrum. Then, we extracted the maximum value along with its corresponding frequency, mirroring the intent of their approach while simplifying the execution.

The process of extracting 13 features from the MFCC components was also unclear. Lacking detailed instructions, we assumed this step involved calculating the mean of each of the 13 MFCC components. Due to a lack of information on how the MFCC features were computed, we used the `librosa`[3] library for our implementation.

Lastly, it was unclear whether hyperparameter tuning was performed for the Random Forest model. In the absence of this information, we assumed the default parameters from scikit-learn's `RandomForestClassifier`[4] were used.

2. Seok et al. work

Figure S2 shows the full signal-processing and comparison workflow used by Seok et al. [5] for the re-identification task.

2.1. Description of the approach

The signal is segmented into 6-second windows to extract temporal features. To improve peak detection accuracy, linear trends are removed from each window, eliminating long-term drifts that could distort feature extraction.

---

2 https://numpy.org/devdocs/reference/generated/numpy.fft.rfft.html
3 https://librosa.org/doc/0.10.2/generated/librosa.feature.mfcc.html#librosa.feature.mfcc
4 https://scikit-learn.org/1.5/modules/generated/sklearn.ensemble.RandomForestClassifier.html

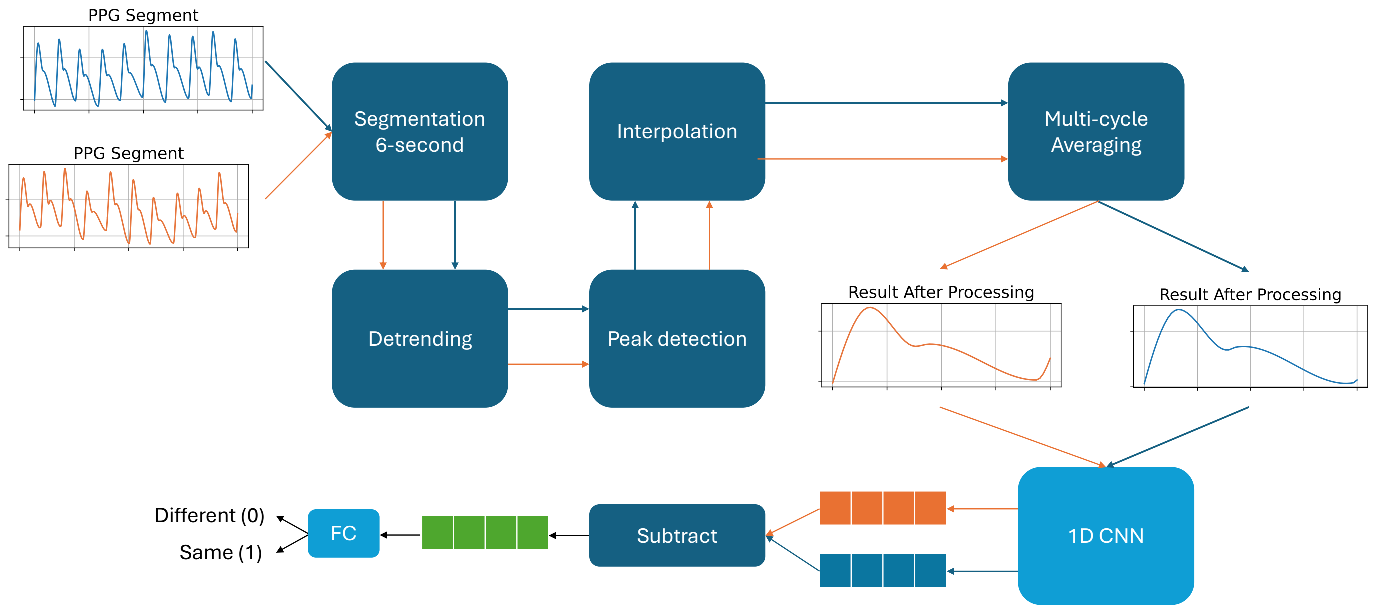

Figure S2. Processing pipeline of the method by Seok et al. [5]. The PPG signal is first segmented into 6-second windows, detrended, and processed through peak detection, quadratic spline interpolation, and multicycle averaging. Each of the two input windows is then processed independently by an identical one-dimensional convolutional neural network (1D-CNN). The resulting feature vectors are subtracted to form a difference vector, which is used to determine whether both windows originate from the same individual.

Feature extraction follows a structured process. Peak detection identifies true peaks while filtering out noise-related artifacts, enabling reliable segmentation into individual cycles. To ensure consistency across samples, quadratic spline interpolation standardizes cycle lengths before averaging. A multicycle averaging technique then overlaps cycles, reinforcing user-specific signal patterns while further reducing noise.

The model is based on a Siamese network with a one-dimensional convolutional neural network (1D-CNN) for feature extraction. After extracting features from two input signals, their corresponding feature vectors are subtracted to compute a difference vector. This vector is then processed by a fully connected layer, which produces a binary classification: 0 for impostors and 1 for genuine users.

Evaluation is performed using the predefined train–test split from the RWPPG dataset [6].

2.2. Issues in the reimplementation

In our efforts to reimplement the methodology from Seok et al. [5], we faced several challenges that necessitated specific choices, as explained below.

First, to detrend the signal, the authors proposed subtracting a moving average with a specified window size (distinct from the one used for segmentation). However, they did not specify the exact window size applied. Without this information, we decided to use SciPy's `detrend`[5] function, which does not require specifying a window size.

Another challenge arose with peak detection. The authors used a threshold to filter out false peaks based on the minimum heart rate. However, they did not provide the specific threshold value. Additionally, we reasoned that the threshold should be set according to the maximum heart rate rather than the minimum. Since a higher heart rate results in shorter distances between true peaks, setting a threshold based on the maximum heart rate would

[5] https://docs.scipy.org/doc/scipy/reference/generated/scipy.signal.detrend.html

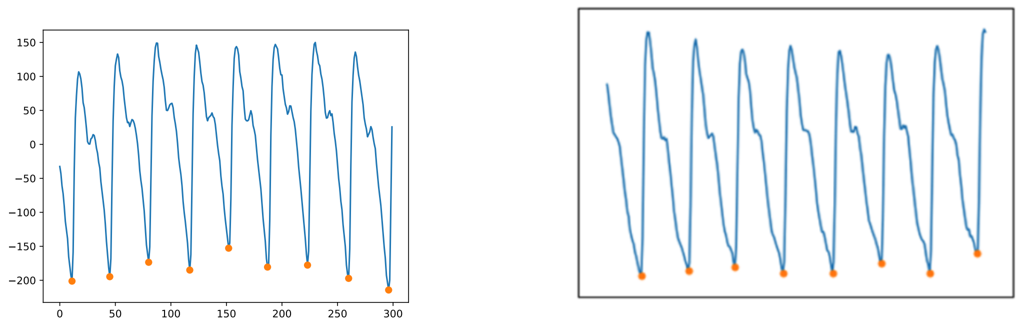

(a) Output of the first input in the test set, highlighting the peaks identified after the false peak removal step.

(b) Output of a sample from the original paper after the false peak removal step.

Figure S3. Comparison of the false peak removal step applied to two similar inputs. (a) Output from our implementation. (b) Output as presented in the original paper.

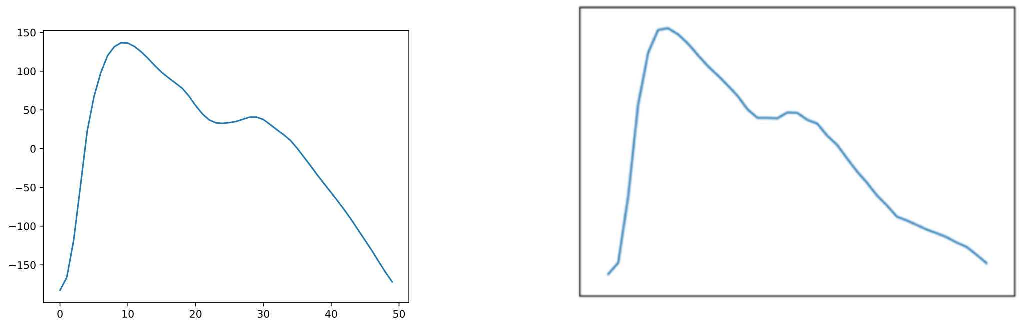

(a) Output of the first input in the test set after single-cycle extraction.

(b) Output of a sample from the original paper after single-cycle extraction.

Figure S4. Comparison of the single-cycle results for two similar inputs. (a) Output from our implementation. (b) Output as presented in the original paper.

successfully filter out spurious peaks. Since the datasets were collected during non-intense activity, we set a maximum heart rate of 180 bpm to allow for some variability in light to moderate activity levels. The threshold was computed as $threshold = (60/max\_hr) \cdot s$, where s is the sampling rate and max_hr is set to 180.

Despite this decision, we encountered an additional issue: when two peaks were closer than the threshold distance, it was unclear how to determine which peak to retain and which to discard. To address this, we used SciPy's `find_peaks` function, setting its distance parameter to match our threshold. This reliable function effectively handled peak detection and, by comparing our output with an image from the original paper, we believe our approach closely replicates their results. Since the original paper did not specify which sample was used for their plot, we selected a similar input for comparison. The comparison can be seen in Figure S3.

Interpolation presented its own set of challenges. Although the authors provided a formula for quadratic spline interpolation, they did not specify its parameters, making it difficult to replicate the exact procedure. To work around this, we used SciPy's interpolate function to perform the interpolation[6]. As shown in Figure S4, our final results are similar

[6] https://docs.scipy.org/doc/scipy/reference/generated/scipy.interpolate.interp1d.html

to those in the original study, suggesting that our re-implementation aligns well with their methodology.

Regarding the model, since a Siamese neural network was used, it was necessary to create all possible pairs from the training and test sets. However, this approach would result in a large number of negative pairs (i.e., pairs where the two samples do not match). To maintain balanced training, they selected positive and negative pairs with a probability of 50%. We interpret this as downsampling the negative pairs, to keep balance between the positive and negative pairs. However, no information was provided on the subsampling method used (e.g., random sampling, sampling per user, and user-based sampling). Therefore, we assumed it was performed randomly.

Another unspecified detail pertained to the kernel sizes of the CNN blocks. Although these were not provided, the input and output dimensions of each layer were reported, enabling us to back-calculate the kernel sizes.

Finally, the threshold for converting values between 0 and 1 into binary predictions was not specified. We assumed a threshold of 0.5, with values above 0.5 considered as 1.

3. Our feature-based method

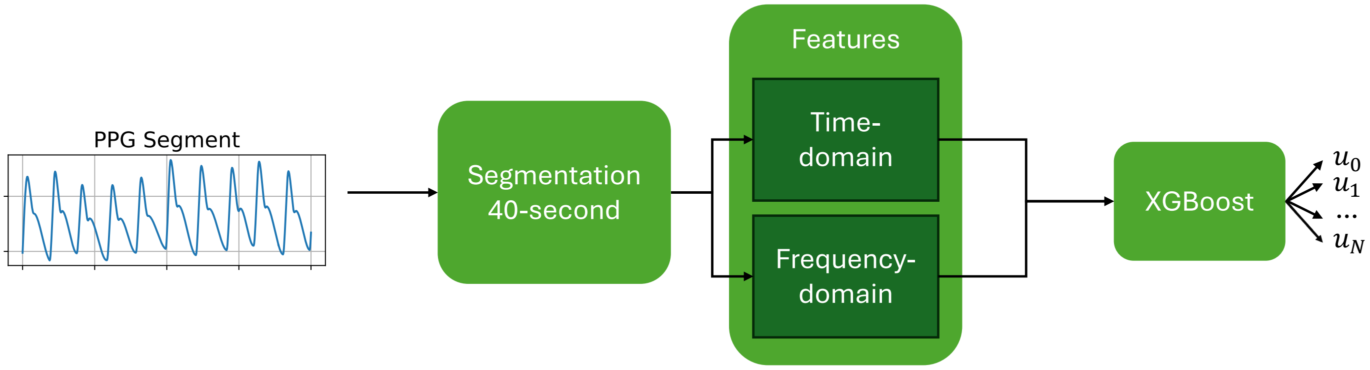

Figure S5. Overview of our feature-based identification method. Each 40-second PPG segment is processed to extract time-domain features (such as amplitude statistics and derivatives) and frequency-domain features (derived from the Fourier transform and Welch spectral estimation). The resulting feature vector is fed into an XGBoost classifier to predict the user's identity.

Figure S5 presents the full workflow of our feature-based method for the user-identification task.

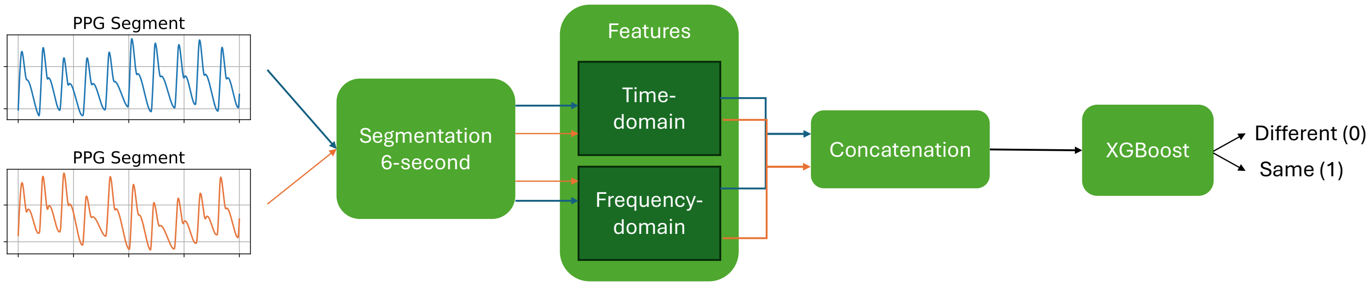

Figure S6. Overview of our feature-based re-identification method. Two 6-second PPG segments are independently processed to extract time-domain and frequency-domain features. The resulting feature vectors are concatenated and provided as input to an XGBoost classifier, which determines whether the two segments originate from the same user or from different users.

Figure S6 illustrates the complete pipeline of our feature-based approach for comparing two PPG windows in the re-identification scenario.

Table S1. List of computed features. All of them can be considered functions $\mathbb{R}^n \rightarrow \mathbb{R}^1$. We define the following conventions: $\overline{x}$ = mean(x), $H(x)$ as Shannon entropy [7] of x, $\max_i x$ as the i-th max, $\arg\max_i x$ as the i-th argmax, and $\mathbb{I}$ as the indicator function. We also define $q_t \in \mathbb{R}^1$ as the t-th value in the signal q.

Name	Formula	Type
Mean	$\overline{q}$	Time-domain
Std	$\sigma(q)$	Time-domain
Min	$\min(q)$	Time-domain
Max	$\max(q)$	Time-domain
Median	$median(q)$	Time-domain
25th Percentile	$p_{25}(q)$	Time-domain
75th Percentile	$p_{75}(q)$	Time-domain
Diff min max	$\max(q) - \min(q)$	Time-domain
Cross mean	$\sum_{t=1}^{n} \mathbb{I}_{(q_t \geq \bar{q}) \neq (q_{t-1} \geq \bar{q})}$	Time-domain
Std $f'(q)$	$\sigma(\nabla_t(q))$	Time-domain
Mean $f'(q)$	$\overline{\nabla_t(q)}$	Time-domain
Mean $f''(q)$	$\overline{\nabla_t^2(q)}$	Time-domain
Std $f''(q)$	$\sigma(\nabla_t^2(q))$	Time-domain
Max abs	$\max_{t \in \{0,\ldots,n\}} \lvert q_t \rvert$	Time-domain
Variance	$\sigma^2(q)$	Time-domain
Energy	$\sum_{t=0}^{n} {q_t}^2$	Time-domain
Entropy	$H(\lvert u \rvert)$	Frequency-domain
Spectral energy	$\sum \lvert u \rvert^2$	Frequency-domain
1st peak value	$\max u$	Frequency-domain
1st peak	$\arg\max u$	Frequency-domain
2nd peak value	$\max_2 u$	Frequency-domain
2nd peak	$\arg\max_2 u$	Frequency-domain
3rd peak value	$\max_3 u$	Frequency-domain
3rd peak	$\arg\max_3 u$	Frequency-domain

For a given window size w_s and sampling rate s, the full signal within a single window is represented as $q \in \mathbb{R}^n$, where $n = w_s * s$. Different signal representations are extracted from the raw PPG data to capture a broader range of features. These include the following:

- FFT Representation: the Fast Fourier Transform (FFT) [8] is applied to the raw PPG signal.
- Welch Representation: Welch's method [9] is applied to the raw PPG signal.
- Wavelet Daubechies 1 Representation: the Wavelet Daubechies 1 is extracted from the raw PPG signal.
- Wavelet Daubechies 4 Representation: the Wavelet Daubechies 4 [10] is extracted from the raw PPG signal.
- Wavelet Symlets 4 Representation: the Wavelet Symlets 4 [11] is extracted from the raw PPG signal.

Then, a comprehensive set of 160 features is extracted to capture a diverse range of patterns essential for user recognition. Their detailed description is provided in Table S1. Below is a textual overview.

- Time-Domain Features: sixteen features (the features reported in Table S1 as "Time-domain") are computed for each of the nine signals: raw PPG, FFT (on the absolute value), Welch (on the absolute value), and the two components of each of the three Wavelets (on the absolute value). Thus, $16 \cdot 9 = 144$ features in total.

- Frequency-Domain Features: eight features (the features reported in Table S1 as "Frequency-domain") are derived specifically from the FFT and Welch representations. Thus, $8 \cdot 2 = 16$ features in total.

For classification, XGBoost[12] is used, as it has consistently demonstrated superior performance compared to other machine learning models[13,14]. XGBoost is particularly suited for this task due to its efficiency, scalability, and ability to capture complex patterns, even though improvements over other models may be marginal in some cases. We employ the standard XGBoost parameters (version `2.1.2`) and set `subsample` to 0.9 and `colsample_bytree` to 0.9.

4. Notes on the Siamese Neural Network approach

Siamese Neural Networks (SNNs), while powerful in learning similarity patterns for a given task, face several inherent challenges that can affect their efficiency and applicability [15]. In typical SNN setups, all possible pairs must be created, including both positive pairs (i.e., pairs of the same class) and negative pairs (i.e., pairs of different classes). This approach can quickly cause the dataset to explode in size, growing exponentially, introducing potential risks of overfitting due to an overwhelming number of samples. Additionally, not all pairs contribute equally to learning, and many may be redundant or provide little new information, diluting the ability of the model to generalize effectively.

The imbalance between positive and negative pairs is another major concern in SNNs. To illustrate this, consider an example of a dataset containing data from 28 users, with 10 windows of data per user. The number of possible positive unique pairs within this dataset can be calculated as

$$\frac{n \cdot (n-1)}{2}$$

for each user's windows, where $n = 10$, resulting in $\frac{10 \cdot 9}{2} = 45$ positive unique pairs per user. Across 28 users, this yields $28 \cdot 45 = 1260$ positive pairs. In contrast, the total number of pairs between all data windows (both positive and negative) is: $\frac{(28 \cdot 10) \cdot (28 \cdot 10 - 1)}{2} = 39060$. Thus, positive pairs constitute only about $\frac{1260}{39060} \approx 3.23\%$ of the total pairs. This imbalance complicates the ability of the model to identify patterns in a dataset overwhelmingly populated with negative pairs.

Training time is another hurdle due to the extensive number of pairs required for SNN training. In particular, Seok et al. [5] used a balanced training set by undersampling the negative pairs to match the number of positive pairs. Specifically, the training set comprises 35 users with approximately 40 samples per participant. This results in a number of positive pairs per user of $\frac{40 \cdot (40-1)}{2} = 780$. Since the training set is balanced by design, the total number of pairs in the training set, accounting for both positive and negative pairs across all users, is $2 \cdot 780 \cdot 35 = 54600$. However, Seok et al. [5] kept the test set unbalanced. Given that the RWPPG test set contains 700 samples, the total number of pairs in the test set amounts to $\frac{700 \cdot (700-1)}{2} = 244650$. Training for 500 epochs, as specified in their model, and testing on a set of 244650 pairs required approximately 4 hours on an NVIDIA RTX A6000 GPU with our implementation in PyTorch[7]. In our experiment, both the training and test sets contain approximately 220 samples per user, with 42 users in total. This results in a total number of pairs in the training set of $\frac{2 \cdot 42 \cdot 220 \cdot (220-1)}{2} = 2023560$, which amounts to about $\frac{2023560}{54600} \approx 37$ times the training set size of RWPPG. Meanwhile, the test set comprises $\frac{42 \cdot 220 \cdot ((42 \cdot 220)-1)}{2} = 42684180$, which is approximately $\frac{42684180}{244650} \approx 174$ times the RWPPG test set size. Such a setting would require weeks of computation if applied as is, underscoring the need for more efficient pairing strategies to manage computational demands.

[7] https://pytorch.org/

References

1. Hinatsu, S.; Suzuki, D.; Ishizuka, H.; Ikeda, S.; Oshiro, O. Photoplethysmographic Subject Identification by Considering Feature Values Derived from Heartbeat and Respiration. In Proceedings of the 2020 42nd Annual International Conference of the IEEE Engineering in Medicine & Biology Society (EMBC), Montreal, QC, Canada, 2020; pp. 902–905. https://doi.org/10.1109/EMBC44109.2020.9176311.
2. Temko, A. Accurate Heart Rate Monitoring During Physical Exercises Using PPG. *IEEE Transactions on Biomedical Engineering* **2017**, *64*, 2016–2024. https://doi.org/10.1109/TBME.2017.2676243.
3. Lapitan, D.G.; Rogatkin, D.A.; Molchanova, E.A.; Tarasov, A.P. Estimation of Phase Distortions of the Photoplethysmographic Signal in Digital IIR Filtering. *Scientific Reports* **2024**, *14*, 6546. https://doi.org/10.1038/s41598-024-57297-3.
4. Virtanen, P.; Gommers, R.; Oliphant, T.E.; Haberland, M.; Reddy, T.; Cournapeau, D.; Burovski, E.; Peterson, P.; Weckesser, W.; Bright, J.; et al. SciPy 1.0: Fundamental Algorithms for Scientific Computing in Python. *Nature Methods* **2020**, *17*, 261–272. https://doi.org/10.1038/s41592-019-0686-2.
5. Seok, C.L.; Song, Y.D.; An, B.S.; Lee, E.C. Photoplethysmogram Biometric Authentication Using a 1D Siamese Network. *Sensors* **2023**, *23*, 4634. https://doi.org/10.3390/s23104634.
6. Siam, A. Real-World PPG Dataset, 2019. https://doi.org/10.17632/YYNB8T9X3D.1.
7. Shannon, C.E. A Mathematical Theory of Communication. *Bell System Technical Journal* **1948**, *27*, 379–423. https://doi.org/10.1002/j.1538-7305.1948.tb01338.x.
8. Brigham, E.O. *The Fast Fourier Transform and Its Applications*; Prentice-Hall Signal Processing Series, Prentice Hall: Englewood Cliffs, N.J, 1988.
9. Welch, P.D. The Use of Fast Fourier Transform for the Estimation of Power Spectra: A Method Based on Time Averaging over Short, Modified Periodograms. *IEEE Transactions on Audio and Electroacoustics* **1967**, *15*, 70–73. https://doi.org/10.1109/TAU.1967.1161901.
10. Daubechies, I. *Ten Lectures on Wavelets*; Number 61 in CBMS-NSF Regional Conference Series in Applied Mathematics, Society for Industrial and Applied Mathematics: Philadelphia, Pa, 1992. https://doi.org/10.1137/1.9781611970104.
11. Daubechies, I. Orthonormal Bases of Compactly Supported Wavelets. *Communications on Pure and Applied Mathematics* **1988**, *41*, 909–996. https://doi.org/10.1002/cpa.3160410705.
12. Chen, T.; Guestrin, C. XGBoost: A Scalable Tree Boosting System. In Proceedings of the Proceedings of the 22nd ACM SIGKDD International Conference on Knowledge Discovery and Data Mining, New York, NY, USA, 2016; KDD '16, pp. 785–794. https://doi.org/10.1145/2939672.2939785.
13. Ramdani, F.; Furqon, M.T. The Simplicity of XGBoost Algorithm versus the Complexity of Random Forest, Support Vector Machine, and Neural Networks Algorithms in Urban Forest Classification, 2022, [11:1069]. https://doi.org/10.12688/f1000research.124604.1.
14. Didavi, A.B.K.; Agbokpanzo, R.G.; Agbomahena, M. Comparative Study of Decision Tree, Random Forest and XGBoost Performance in Forecasting the Power Output of a Photovoltaic System. In Proceedings of the 2021 4th International Conference on Bio-Engineering for Smart Technologies (BioSMART), Paris, France, 2021; pp. 1–5. https://doi.org/10.1109/BioSMART54244.2021.9677566.
15. Li, Y.; Chen, C.L.P.; Zhang, T. A Survey on Siamese Network: Methodologies, Applications, and Opportunities. *IEEE Transactions on Artificial Intelligence* **2022**, *3*, 994–1014. https://doi.org/10.1109/TAI.2022.3207112.

Disclaimer/Publisher's Note: The statements, opinions and data contained in all publications are solely those of the individual author(s) and contributor(s) and not of MDPI and/or the editor(s). MDPI and/or the editor(s) disclaim responsibility for any injury to people or property resulting from any ideas, methods, instructions or products referred to in the content.
